# Supplementary material for: Multi-Omic Analysis Identifies Key Genes Driving Testicular Fusion in Spodoptera litura
Source: Int J Mol Sci. 2025 Jun 10;26(12):5564. doi: 10.3390/ijms26125564 (PMC12193397; doi:10.3390/ijms26125564)
Supplement: Supplementary file 1 [file ijms-26-05564-s001.zip › Supplementary figure and table captions.pdf]

Figure legends

Figure S1. Violin plots illustrating the gene expression accounts in the testicular sheath at pre-fusion (*Sl*_L6D3), mid-fusion (*Sl*-PP), and post-fusion (*Sl*-P3) stages.
Figure S2. (A) GO enrichment of up regulated proteins during fusion vs pre-fusion period. (B) GO enrichment of down regulated proteins during post-fusion vs fusion period.
Figure S3. Co-expression Gene and Protein GO Enrichment Analysis. (A) Genes and proteins that are upregulated compared between fusion vs prefusion. (B) Genes and proteins that are downregulated compared between postfusion vs fusion.
Figure S4. Comparison of the temporal expression profiles of five selected genes in the testes of the *B.mori* and *S.litura.*
Figure S5. Design and validation of *Sl3030* sgRNA target sites. (A) Gene structure of *Sl3030.* (B) The three-dimensional structure and transmembrane region of *Sl*3030. (C) The disordered region structure of *Sl*3030. (D) The subcellular localization of *Sl*3030.(E) Sequence similarity comparison of the unknown function gene Sl3030 in Lepidoptera and other model organisms
Figure S6. (A) Schematic diagram of the gene structure of Sl3030 showing the positions of exons and gRNAs.(B) Bimodal graph of the G0 generation mutant.(C) Genotype identification of the G2 generation homozygous mutant. (D) The secondary structure of the protein in the mutant is different from that in the wild type. (E) Differences in spatial structure between the mutant and the wild type.
Figure S7. The effect of *Sl3030* gene knockout on testicular morphology and spermatogenesis. (A) Morpholgy of wild-type and mutant testis under an optical microscope. (B) Comparison of testis sections between wild-type and mutant. (C) Histogram illustrating testis sizes of wild type and mutant. (D) Comparison of the testis internal structure between wild-type and mutant testes. The red arrow points to the delicate follicular epithelium.
Figure S8. KEGG enrichment analysis of down regulated genes in the mutant.
Table S1. PCR amplification primers in this article.
