# Supplementary material for: Multi-Omic Analysis Identifies Key Genes Driving Testicular Fusion in Spodoptera litura
Source: Int J Mol Sci. 2025 Jun 10;26(12):5564. doi: 10.3390/ijms26125564 (PMC12193397; doi:10.3390/ijms26125564)
Supplement: Supplementary file 1 [file ijms-26-05564-s001.zip › Table S1.pdf]

Primer name	Sequence (5'-3')
Primers for sgRNA	
Sl3030-sgRNA-F-1	TAATACGACTCACTATAGGTCCACCTGCGCCAACTTAG TTTTAGAGCTAG AAATAGCAAGTTAAAATAAG
Sl3030-sgRNA-F-2	TAATACGACTCACTATAGGCACATAGTTACGGGCCCCG TTTTAGAGCTAGAAATAGCAAGTTAAAATAAG
Primers for cloning DNA templates of sgRNA transcription	
18T-F	CGGTGATGACGGTGAAAACCTC
18T-R	AAGCACCGACTCGGTGCC
Primers for mutant screening	
Sl3030-Test-F	GATTACTCGCCGCCGTTTTC
Sl3030-Test-R	AAAGCTTCGTTCGTCCTCGT
Primers for q-RTPCR	
Sl3030--F	GACCATCAAACGGAACGAAA
Sl3030--R	AACGGCGGCGAGTAATCAT
