# Supplementary figures and images for: Multi-Omic Analysis Identifies Key Genes Driving Testicular Fusion in Spodoptera litura
Source: Int J Mol Sci. 2025 Jun 10;26(12):5564. doi: 10.3390/ijms26125564 (PMC12193397; doi:10.3390/ijms26125564)

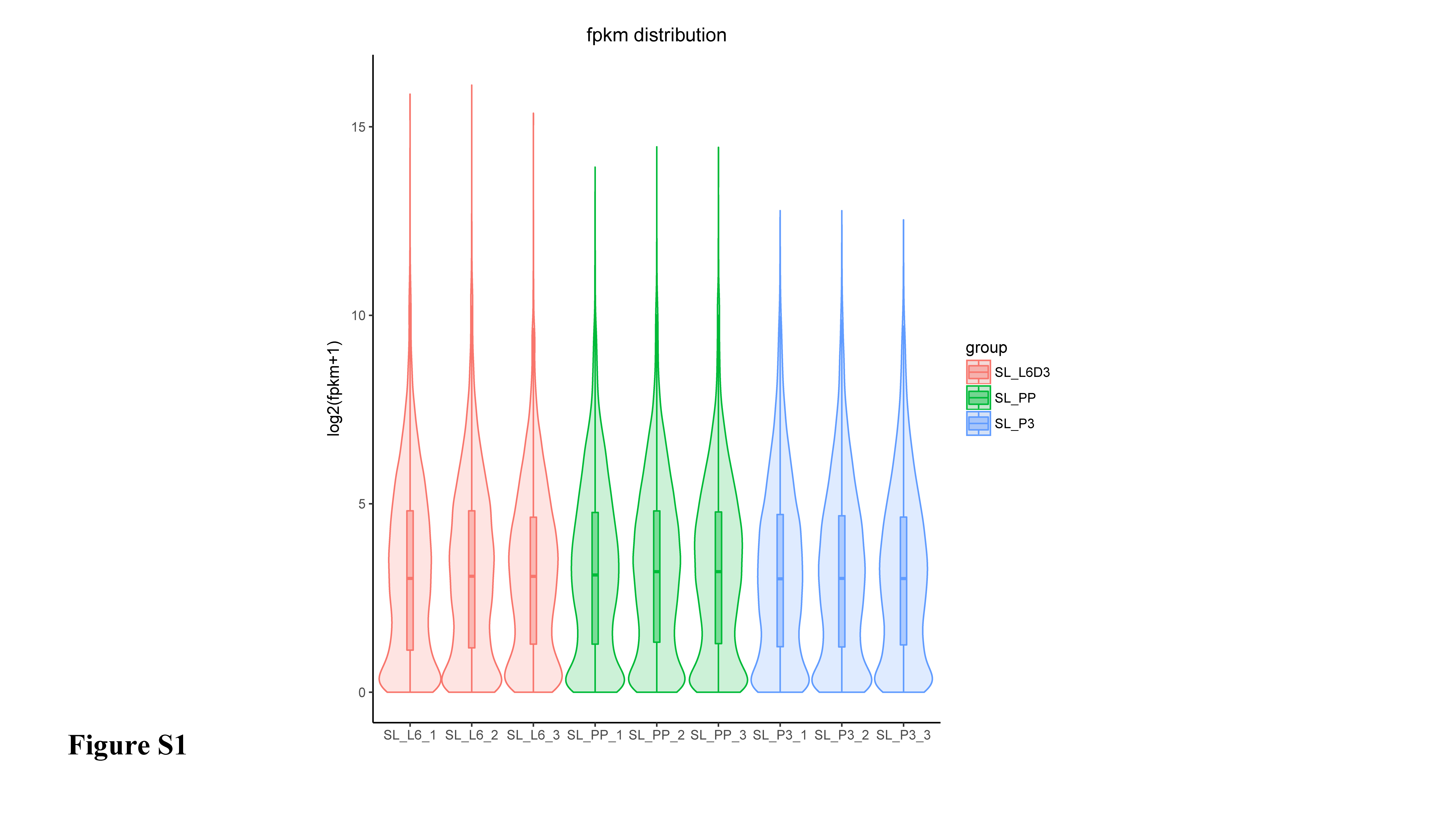

Supplement: Supplementary file 1 [file ijms-26-05564-s001.zip › Figure S1.tif]

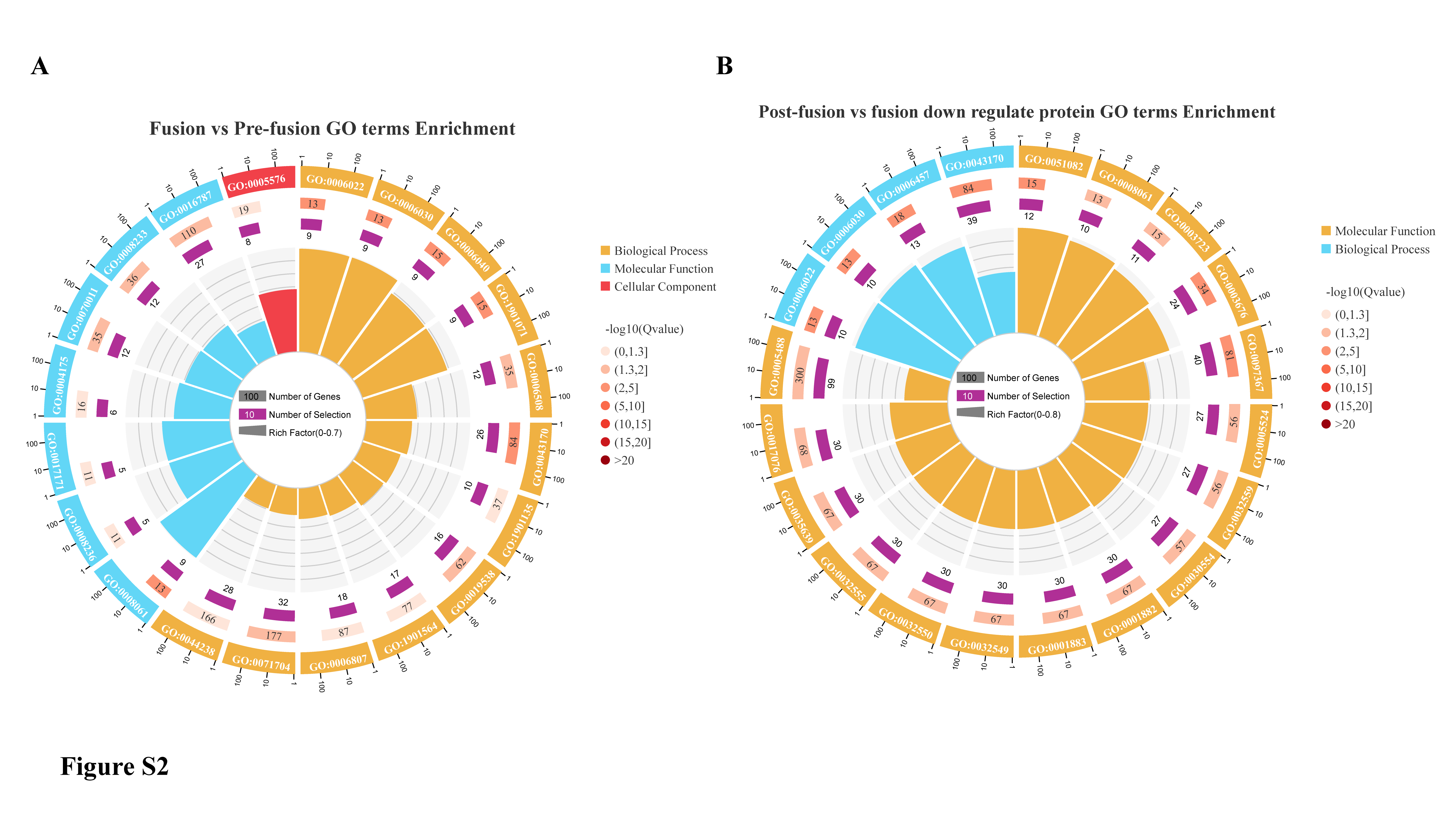

Supplement: Supplementary file 1 [file ijms-26-05564-s001.zip › Figure S2.tif]

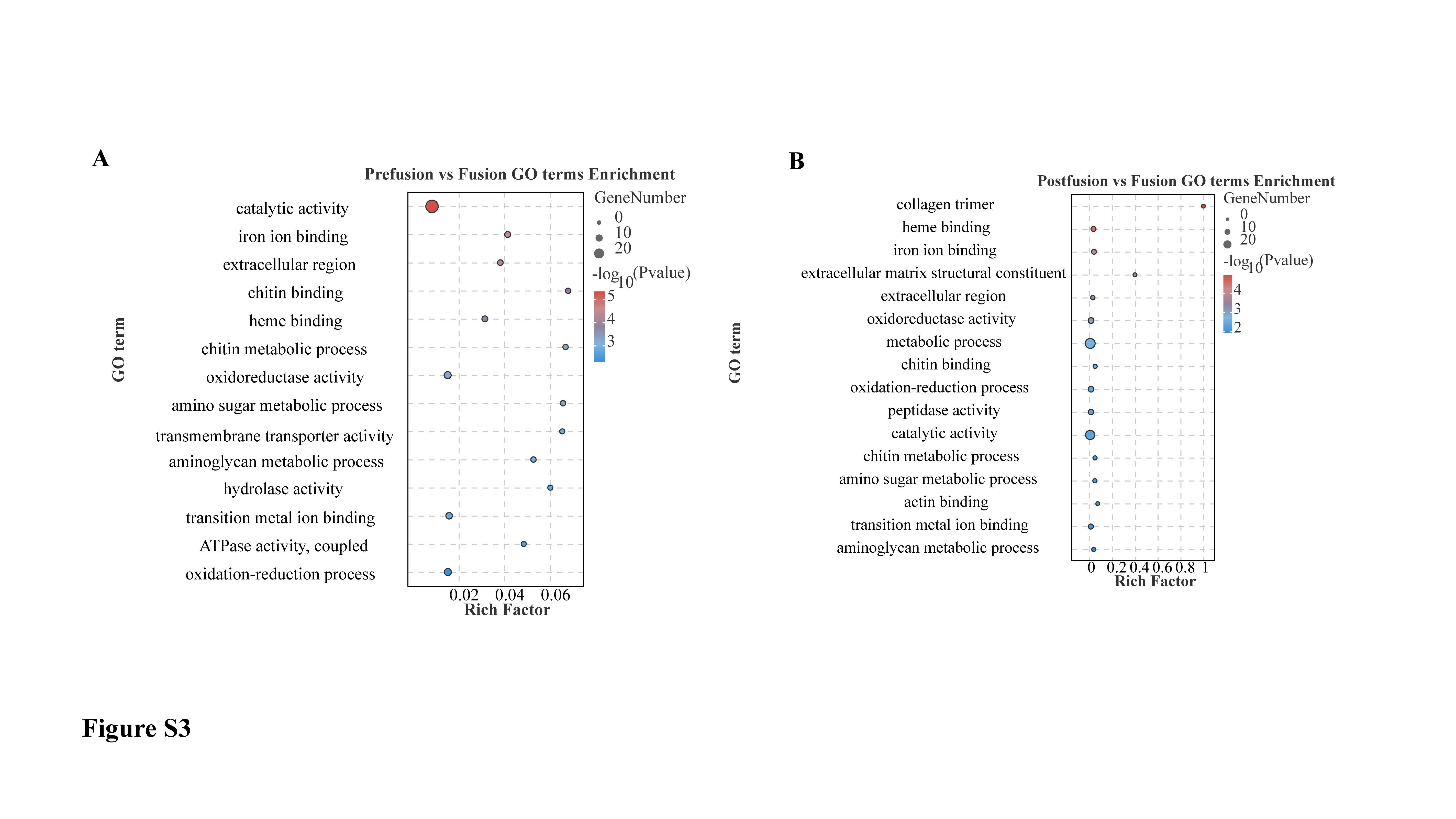

Supplement: Supplementary file 1 [file ijms-26-05564-s001.zip › Figure S3.tif]

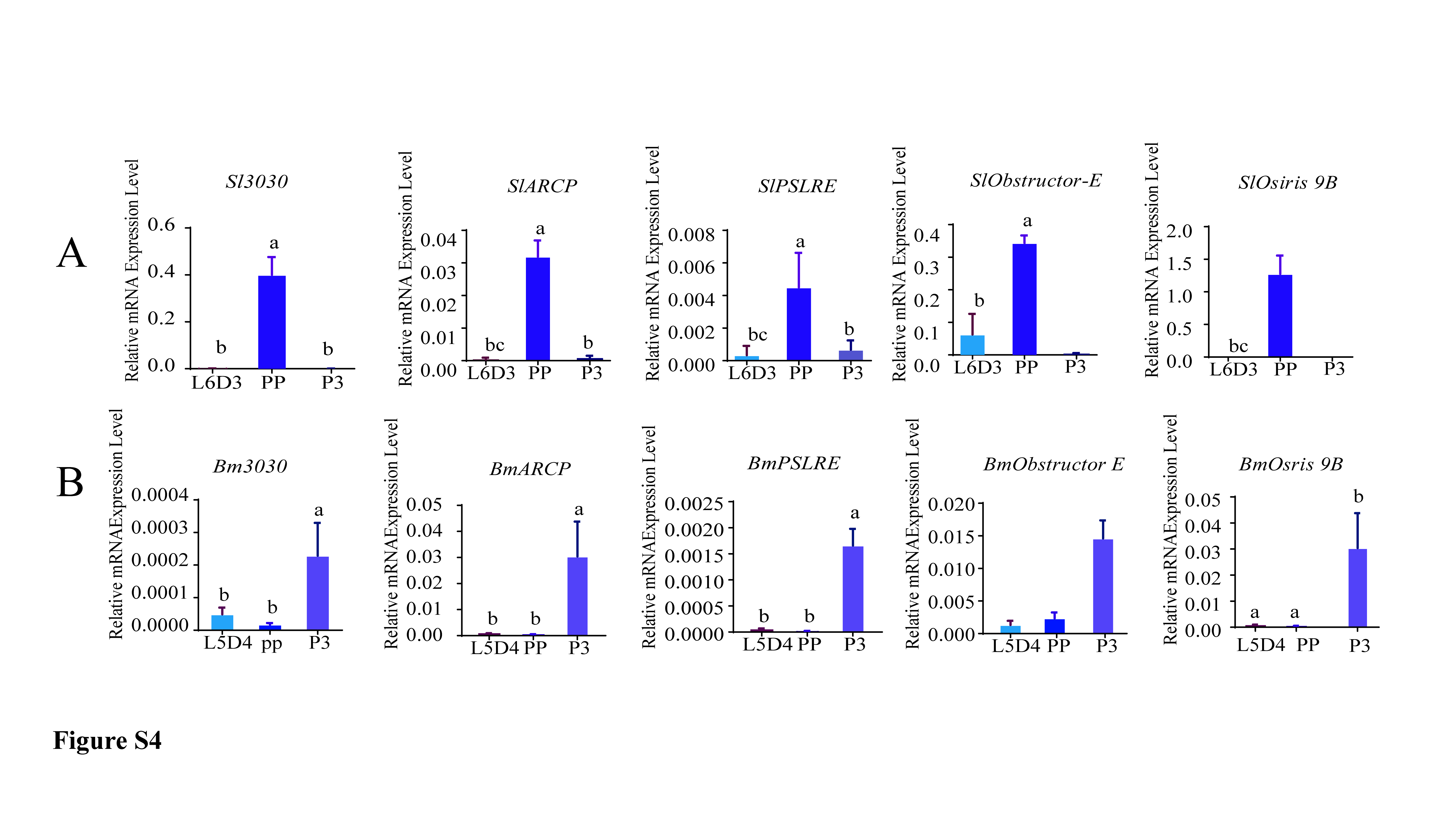

Supplement: Supplementary file 1 [file ijms-26-05564-s001.zip › Figure S4.tif]

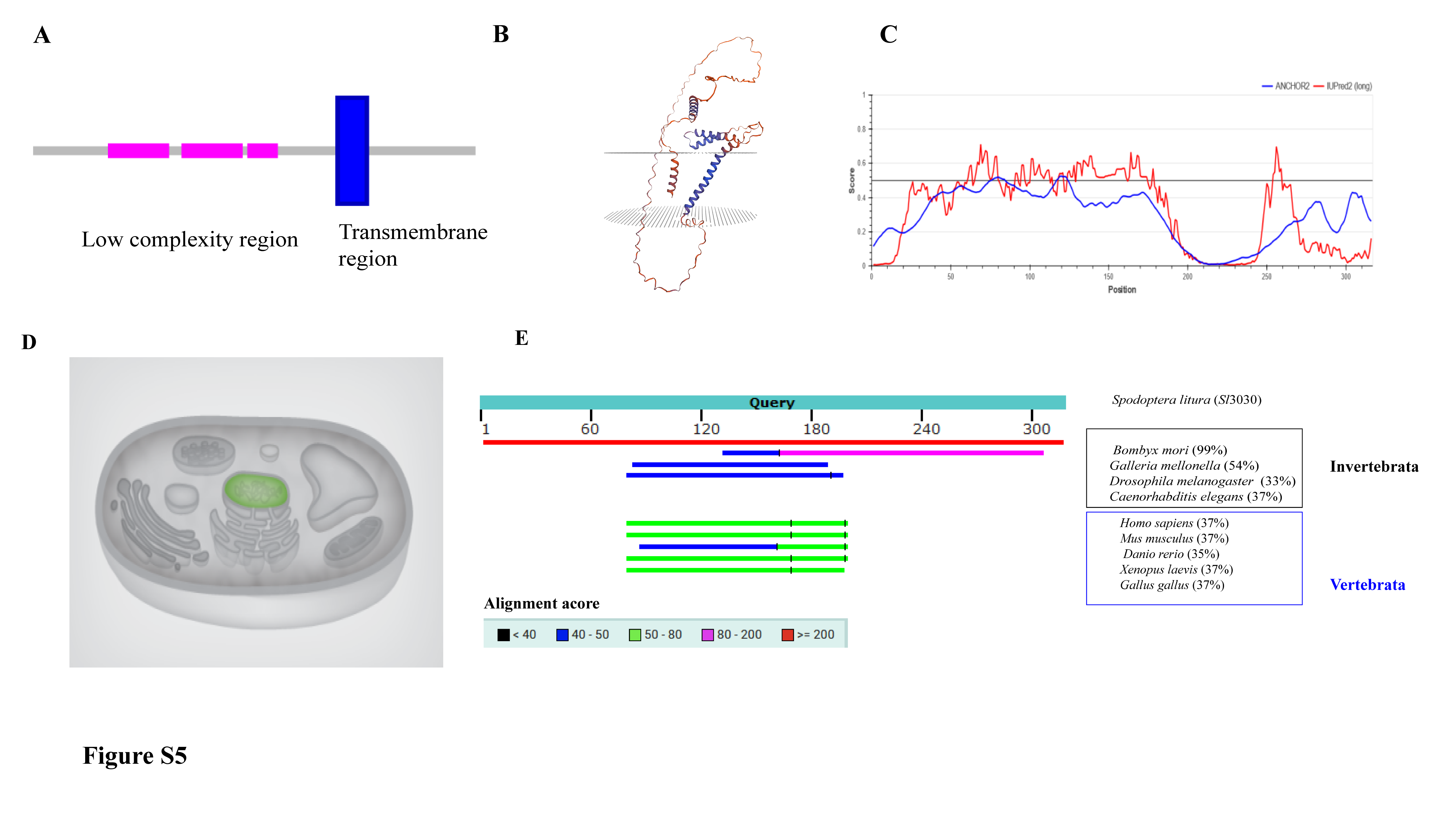

Supplement: Supplementary file 1 [file ijms-26-05564-s001.zip › Figure S5.tif]

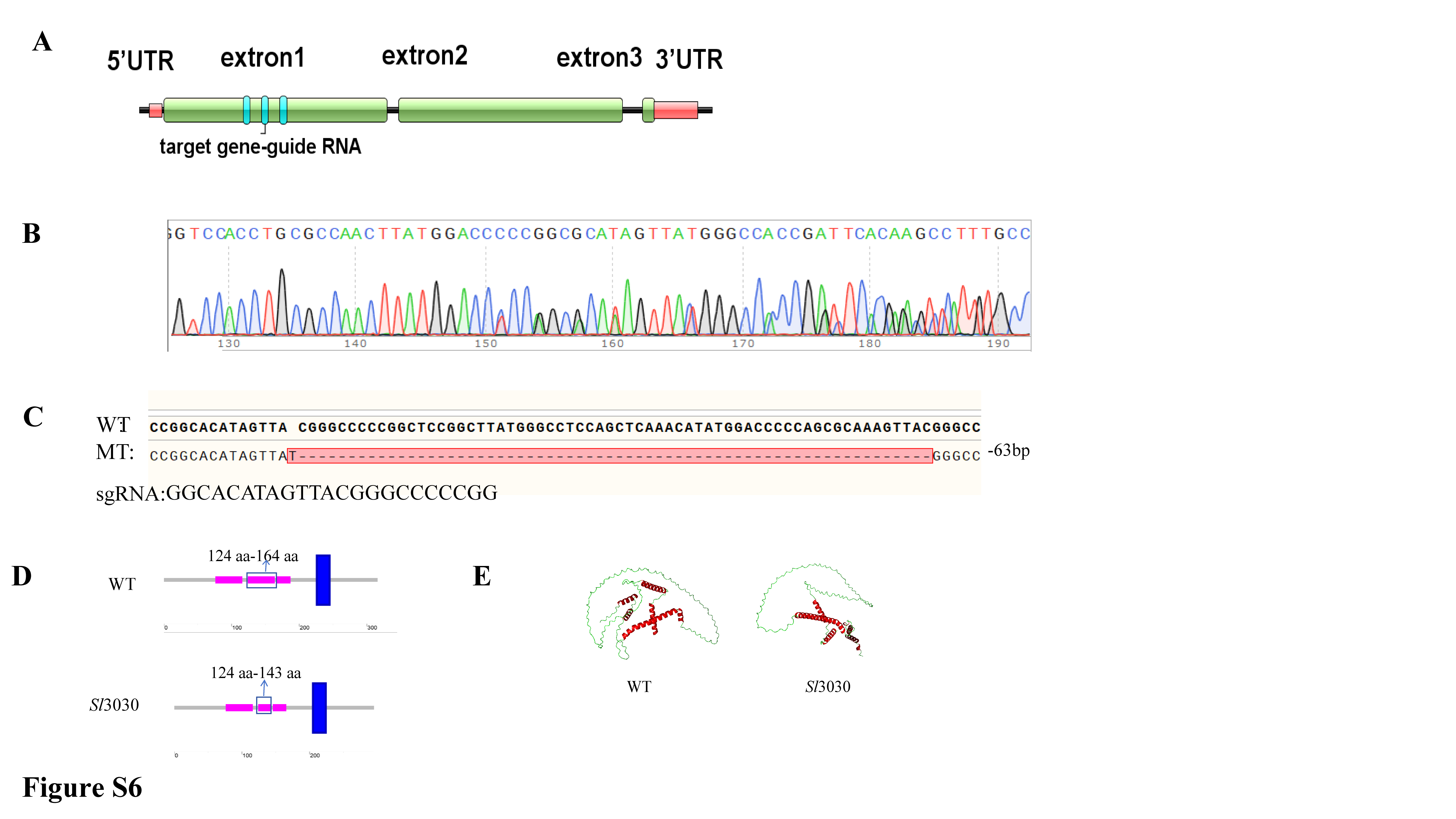

Supplement: Supplementary file 1 [file ijms-26-05564-s001.zip › Figure S6.tif]

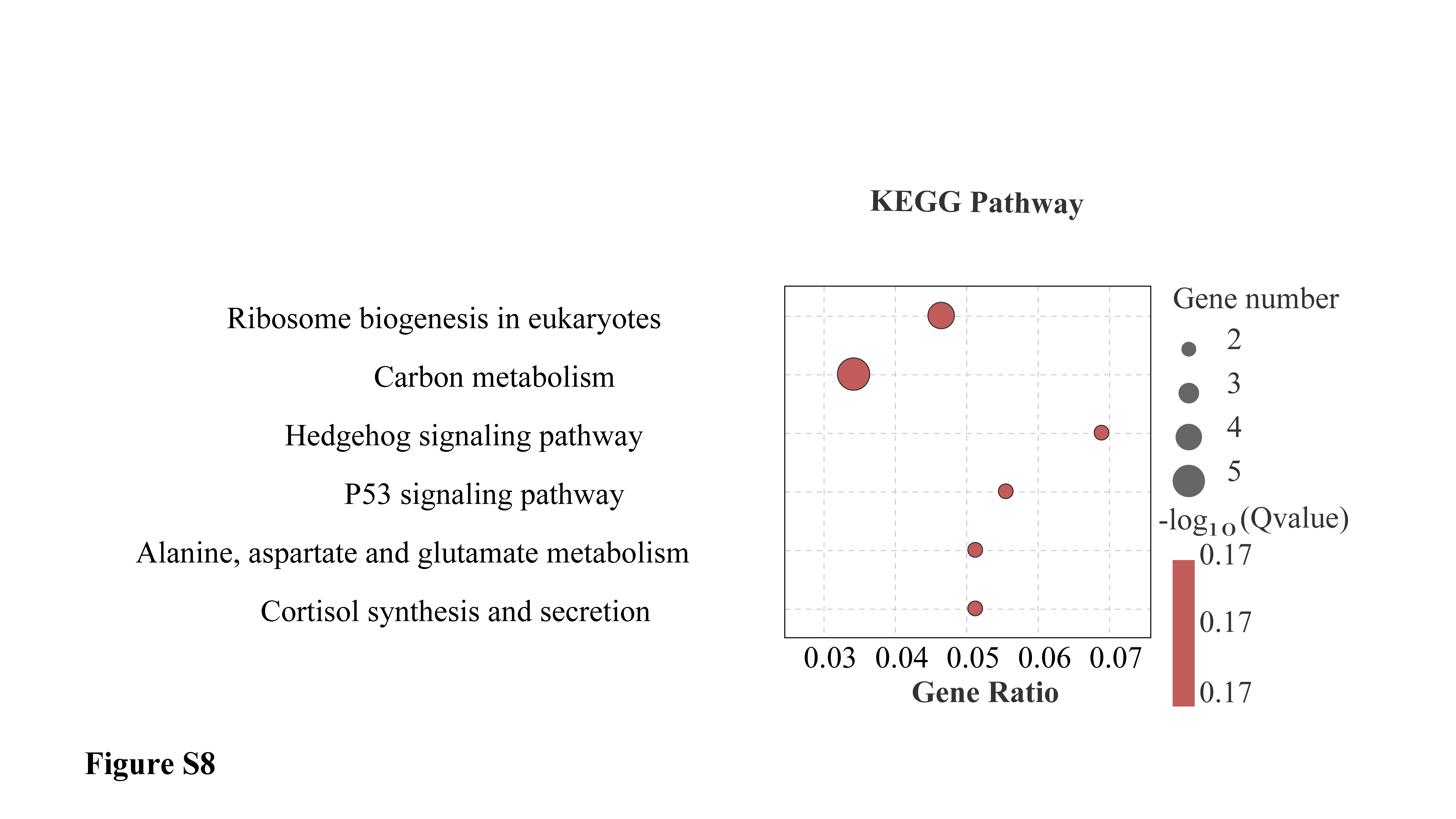

Supplement: Supplementary file 1 [file ijms-26-05564-s001.zip › Figure S8.tif]
